# Preliminary Studies on Detection of Fusarium Basal Rot Infection in Onions and Shallots Using Electronic Nose

**DOI:** 10.3390/s22145453

**Published:** 2022-07-21

**Authors:** Malgorzata Labanska, Sarah van Amsterdam, Sascha Jenkins, John P. Clarkson, James A. Covington

**Affiliations:** 1 The Plant Breeding and Acclimatization Institute-National Research Institute, Radzikow, 05-870 Blonie, Poland; 2 Warwick Crop Centre, School of Life Sciences, University of Warwick, Wellesbourne, Warwick CV35 9EF, UK; sarah.van-amsterdam@warwick.ac.uk (S.v.A.); sascha.jenkins.1@warwick.ac.uk (S.J.); john.clarkson@warwick.ac.uk (J.P.C.); 3 School of Engineering, University of Warwick, Coventry CV4 7AL, UK

**Keywords:** electronic nose, onions, *Fusarium oxysporum*, plant pathogen detection, volatile organic compounds, fungal disease, pattern recognition

## Abstract

The evaluation of crop health status and early disease detection are critical for implementing a fast response to a pathogen attack, managing crop infection, and minimizing the risk of disease spreading. *Fusarium oxysporum* f. sp. *cepae*, which causes fusarium basal rot disease, is considered one of the most harmful pathogens of onion and accounts for considerable crop losses annually. In this work, the capability of the PEN 3 electronic nose system to detect onion and shallot bulbs infected with *F. oxysporum* f. sp. *cepae*, to track the progression of fungal infection, and to discriminate between the varying proportions of infected onion bulbs was evaluated. To the best of our knowledge, this is a first report on successful application of an electronic nose to detect fungal infections in post-harvest onion and shallot bulbs. Sensor array responses combined with PCA provided a clear discrimination between non-infected and infected onion and shallot bulbs as well as differentiation between samples with varying proportions of infected bulbs. Classification models based on LDA, SVM, and k-NN algorithms successfully differentiate among various rates of infected bulbs in the samples with accuracy up to 96.9%. Therefore, the electronic nose was proved to be a potentially useful tool for rapid, non-destructive monitoring of the post-harvest crops.

## 1. Introduction

In a background of an intensive global market, climate change, and countless people and goods travelling all over the world, the spread of disease on a large scale is an inevitable threat. A recent epidemic event has highlighted the hazard and severity of such a scenario [1]. Pathogen-related crop losses result in significant implications for global food production and food security. It is reported that annually up to 40% of crop production is damaged due to weeds, pests, and pathogens, resulting in significant reductions in the quality and quantity of crop yields [2,3]. 

One of the major causes of plant diseases are fungal pathogens, which accounts for 70–80% of infections [4]. Fungal propagules can remain dormant for a long time until favourable conditions for their germination and proliferation occur. Among the top 10 fungal pathogens, *Fusarium oxysporum* is one of the most important in terms of scientific and economic impact. *F. oxysporum* is a soil-borne pathogen and, at a species level, has a wide range of hosts, including tomatoes, melons, beans, onions, bananas, and cotton. However, the individual specific forms (*formae speciales*) of this species complex are highly host-specific and only infect one or a few plant species [5,6]. *F. oxysporum* f. sp. *cepae*, which causes fusarium basal rot disease, is considered one of the most harmful pathogens of onion crops. It has been reported that *ca*. 30% of the onions’ losses are related to *Fusarium* spp. Considering that the annual world production of dry onions in 2020 was around 104.5 million tonnes and was valued *ca*. $44 billion (FAOSTAT, 2022), these losses are significant [7]. The pathogen can affect onion plants at any stage of development, but the disease is most commonly observed in the bulbs. The biggest losses occur when the fungus has infected onion bulbs without visible symptoms close to harvest, which once placed in storage facilities, develop basal rot that may spread to other bulbs. In some cases, this can cause the loss of the entire store of onions.

The evaluation of crop health status and early disease detection are critical for implementing crop and store management responses to minimize impact and further disease spread. Traditional methods for assessment of plant fungal diseases include examination and interpretation of visual symptoms, microbiological identification, and biochemical characterization. However, results from these approaches are often too late to implement control measures and hence new techniques involving antigen-antibody interaction and polymerase chain reaction (PCR) have been introduced in laboratories all over the world [4]. Recently, complementary to these molecular approaches, new tools for plant disease detection have been proposed. These include spectroscopic techniques for plant stress profiling and gas chromatography-mass spectrometry (GC-MS) along with the electronic nose (EN) for profiling plant volatile organic compounds (VOCs) [8,9]. Volatile substances have been associated with plant defence against pathogen attack, warning the neighbouring plants and facilitating reproduction by attracting pollinators and seed dispersers. In addition, the breakdown of plant tissues upon pathogen infection may also result in characteristic VOC profiles [10]. VOCs, therefore, carry a considerable amount of information about the plants’ health status and the stresses to which they have been subjected [11]. Furthermore, the fungus itself can also emit VOCs due to its metabolic activity. As a result, VOC-based diagnostic methods have been intensively investigated since they offer non-destructive, in-field measurements without the need for sample preparation and pathogen isolation and also offer the possibility of simultaneous detection of several pathogens. GC-MS has been widely applied to detect plant-emitted VOCs. A number of papers have discussed its considerable capabilities of detecting pathogen-related chemicals released following infection of potatoes [12], apples [13,14], kiwifruit plants [15], melon fruits [16], wheat [17], and oil palm wood [18] among others. 

There are however disadvantages of most current MS-based (mass-spectrometry-based) systems, including the requirement for trained users, expense, and the fact that they are not sufficiently robust, portable, or fast for direct in-field analysis. Moreover, successful discrimination between healthy and infected samples relies on finding a specific biomarker, while often differences in emitted VOCs are quantitative and ambiguous. Therefore, electronic noses, which provide differentiation of gaseous samples based on their unique, volatile profiles, are potentially useful tools for plant disease detection and recognition, before visual symptoms occur [19,20]. 

Introduced as an attempt to mimic the human olfactory system, the electronic nose consists of an array of chemical sensors with partial selectivity and a pattern recognition system capable of discriminating complex odours [21]. A sensor array generates a characteristic “fingerprint” of a sample, due to the fact that each sensor within the array responds uniquely to a sample.

A variety of gas sensors have been used in electronic noses such as metal-oxide semiconductors (MOS), metal-oxide semiconductor field-effect transistors (MOSFET), electrochemical (EC), quartz crystal microbalances (QCM), and surface acoustic wave (SAW) sensors [22,23]. The data produced by the sensor array are processed using machine pattern recognition algorithms in order to distinguish and classify samples [24,25]. Further description of the electronic nose technology can be found elsewhere [26,27,28,29].

Over the last four decades, electronic noses have been developed and extensively used in a broad range of applications including food analysis [30,31,32,33,34,35], environmental monitoring [36], disease diagnostic [37], and detection of explosives [38]. Electronic noses have also shown great potential in addressing agricultural and forestry challenges, namely detecting plant and trees infections, identifying pest infestations, controlling food production and storage systems, and assessing trees and plants’ physiological state [20,39,40]. Successful applications of commercial devices and laboratory prototypes of an electronic nose for detecting fungal infections in the growth medium [41], strawberry fruit [42], garlic [43], wheat [44], apples [45], and peaches [46] have been reported. Despite a series of works by Li et al. [47,48,49] that focused on the detection of post-harvest bacterial onion diseases, to the best of our knowledge, fungal onions and shallots infections have not previously been studied using an electronic nose. Therefore, the aim of this study was to investigate the capability of an electronic nose to detect onion and shallot bulbs infected with *Fusarium oxysporum* f. sp. *cepae*, track the development of fungal infection, and discriminate between the varying proportions of infected onion bulbs. Furthermore, the influence of storage temperature on the rate of development of fusarium basal rot disease was also studied.

## 2. Materials and Methods

### 2.1. Sample Preparation

Forty healthy onions and twenty shallots were obtained from Parrish Farms (FB Parrish & Sons Ltd., Bedfordshire, UK). Bulbs were subjected to a thorough visual examination to ensure the absence of any disease, decay, and mechanical or microbial damage to ensure they were healthy. This involved checking for the presence of any fungal mycelium on the surface and checking that the tissue around the basal plates was firm with no softness which would have indicated the presence of an existing internal rot which would have affected the experimental results. Twenty onion bulbs and ten shallots were then inoculated with *Fusarium oxysporum* f. sp. *cepae* (FOC) isolate FUS2 as described by Taylor et al. [50]. The outer dry scales of the bulbs were removed, and a 2–3 mm slice of the basal plate was removed with a scalpel, before spraying the bulbs with 70% ethanol. Once dry, an 8 mm plug of potato dextrose agar (PDA) containing the actively growing edge of a FOC isolate FUS2 colony was inverted and placed on the cut basal plate of each bulb. Control, non-inoculated treatments were also prepared in the same way using twenty onion bulbs and ten shallot bulbs, but no agar plug was placed on the basal plate. Bulbs were first placed in damp boxes in sealed plastic bags and incubated at 20 °C for 24 h. After this initial incubation, the non-inoculated and inoculated bulbs were placed in the experimental system which consisted of sealed 3 L plastic containers with inlet and outlet fittings added at both ends. These containers had previously been repeatedly washed to remove any contaminants from the manufacturing process.

**Figure 1:**
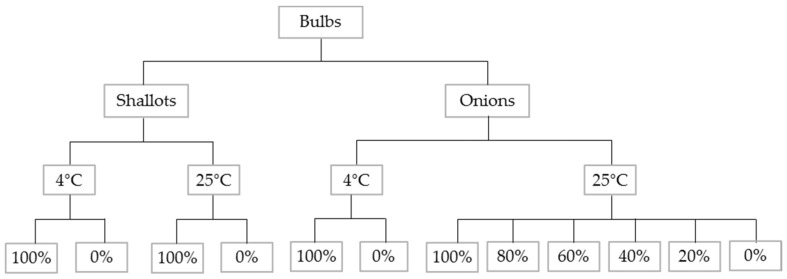
Schematic representation of the tested samples’ set.

The main aims of this work were (a) to study the differences between varying proportions of inoculated to non-inoculated bulbs, (b) to assess the influence of the temperature storage on the disease progression, and (c) to identify infected shallots and onions, based on volatile chemical compounds released by bulbs. Therefore, the set of 12 samples consisted of 3 subsets to deal with these tasks. Samples consisted of healthy and inoculated shallot or onion bulbs (5 in total) at varying proportions, i.e., 0%, 20%, 40%, 60%, 80%, and 100%, in the containers which were stored at two different temperatures resulting in 12 samples. Samples were prepared and stored at 25 °C and 4 °C for 5 weeks and 9 weeks, respectively. The temperature of 25 °C was selected to allow adequate development of basal rot disease over the time course of the experiment but also to reduce the risk of the potential of bacterial rots which can sometimes occur at higher temperatures. Since a higher temperature facilitates disease progression, creating strong signals quicker, varying proportions of the inoculated to non-inoculated bulbs, stored only at 25 °C, were investigated. To ensure that *Fusarium* infection developed, high humidity was maintained in the containers throughout the experiment by placing the bulbs on a plastic rack over deionised water. The humidity in each container was at a similar level. As visible symptoms of the infections were observed in the first week of the experiment, the humidity was at the level providing optimal conditions for infection. The setup of the studied samples is shown in Figure 1.

### 2.2. Electronic Nose Measurements

In this study, the compounds released by the onion and shallot bulbs were analysed by a PEN 3 electronic nose system (Airsense Analytics GmbH, Schwerin, Germany) equipped with 10 MOS sensors, which exhibit differential sensitivity to classes of inorganic and volatile compounds. The general description of the PEN 3 sensor array is summarized in Table 1 [42,45].

**Figure 2:**
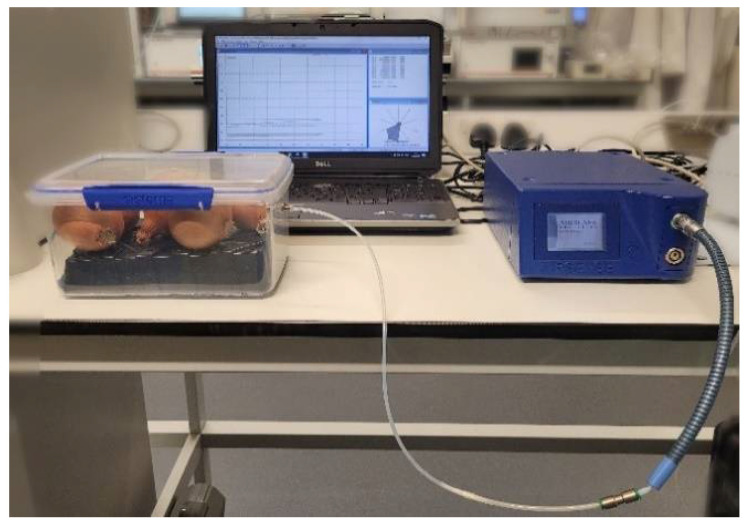
Laboratory setup for the measurements of volatiles released by bulbs using PEN 3 electronic nose system.

In the experiments, the electronic nose’s inlet sampler was connected to the fitting in the sample container and the headspace above the bulbs was pumped at a constant rate of 400 mL/min and injected into the sensor chamber (Figure 2). MOS sensor responses are a result of the interaction between the compounds in the headspace and the metal-oxide semiconductor surface. Recorded signals are the ratios (G/G_0_) of the conductivity of a sensor exposed to a sample (G) and to clean air (G_0_).

The measurements were carried out twice a week, for 5 weeks for those samples stored at 25 °C and 9 weeks for those samples stored at 4 °C. Prior to the measurements, the containers stored at 4 °C were placed at 25 °C to reduce the influence of the temperature on the sensors.

Sensors signals were collected for 180 s at 2 s intervals. After each measurement, the sensor chamber was cleaned with filtered air for 60 s, and the baseline was set for 10 s prior to the following measurement. The responses of the 10-sensor array provided a characteristic pattern for each studied gas mixture released by the sample.

### 2.3. Data Analysis

The instrument saves the data in a relative ratio of G/G_0_, therefore, the maximum or minimum value of G/G_0_ was used as the feature. The max. and min. were selected based on visual inspection of the direction of response.

Principal component analysis (PCA) was applied to explore the EN’s data and reduce its dimensionality without loss of information. It is based on calculating new orthogonal principal components (PCs), which are linear combinations of the original variables. Here, the first few components containing the most information (explaining the highest variance of the data) form axes on 2– or 3–D graphs [51,52]. Several supervised methods, namely linear discriminant analysis (LDA), k-nearest neighbours (k-NN), and support vector machine (SVM), were employed for the samples’ classification. Evaluation of the classifiers’ performance was based on accuracy, recall, and F1 score [53]. The prediction capability of the models was evaluated by using a 5-fold cross-validation to determine its reliability. LDA is a well-known method that involves calculating linear discriminant functions that maximize the inter-class variance and minimize the inter-class variance [54]. K-NN is one of the most commonly applied chemometric tools for data analysis particularly due to its simplicity and intuitive interpretation [55]. SVM algorithm was introduced by Vapik et al. [56] in the 1990s and has since proved to be a useful tool for pattern recognition. Data processing was carried out in MATLAB R2020a (The Math-Works Inc., Natick, MA, USA) and Origin (OriginLab Corporation, Northampton, MA, USA) software.

## 3. Results

### 3.1. Visual Examination of the Fusarium Basal Rot Symptoms

Following the electronic nose measurements, onion and shallot bulbs were visually examined for disease symptoms throughout the experiment. Severe symptoms included the rotting of the entire bulb basal plate, the occurrence of white mycelium on the outside of the bulbs and basal areas of exterior bulb scales, and loss of tissue firmness [57]. Visual inspection of the samples revealed gradual development of these symptoms in inoculated onion and shallot bulbs during the experiment as illustrated in Figure S1.

The first symptoms of Fusarium infection occurred after one week for the inoculated onion and shallot bulbs stored at 25 °C and these continued to develop more severe disease over the course of the experiment. In contrast, inoculated bulbs stored at 4°C remained apparently healthy and this was also the case for non-inoculated control bulbs. At the end of the experiment, all bulbs were dissected to more accurately assess internal rot due to Fusarium basal rot disease after 31 days for those stored at 25 °C and 63 days for those at 4 °C. Here, distinct differences were observed between the inoculated onion and shallot bulbs stored at these two temperatures. All of the bulbs stored at 25 °C developed extensive internal rot with the bulb tissue soft and watery with a dark brown appearance particularly at the basal plate and extending forwards in the bulb, while those stored at 4 °C had no disease at all (Figure S2). These results are consistent with a report that Fusarium basal rot infection is favoured by higher temperatures, with a reported optimum of 28–32 °C with temperatures below 12 °C leading to little or no infection [58].

Finally, while all Fusarium inoculated onion and shallot bulbs developed severe basal rot at 25°C, the uninoculated (control) bulbs remained disease free. This demonstrated that there was no background disease or contamination of the bulbs used in our experiments and therefore that all the disease observed was due to our artificial inoculation.

### 3.2. Sensor Array Responses

The sensors’ responses to chemicals collected from non-infected control onion bulbs stored at 25 °C for 1.5 weeks post inoculation is shown in Figure S3. Each curve shows the changes in the sensor’s conductivity induced by the adsorption of the chemicals onto the metal-oxide surface of the sensor. The sensors’ responses varied significantly according to its sensitivity. In the initial phase of the measurement, sensor outputs rapidly change, but most of the sensors’ signals were in a steady state after 40 s. The W1S and W6S sensors’ signals gradually increased throughout the measurement, although the increase was negligible in the final phase.

**Figure 3:**
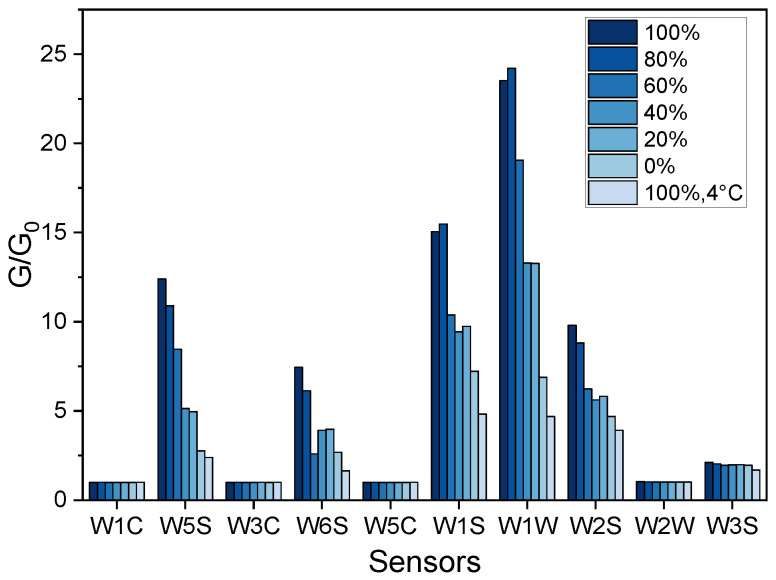
Features extracted from sensor array responses to chemicals emitted by samples consisting of varying proportions of Fusarium-infected onion bulbs stored at 25 °C and samples with 100% infected bulbs stored at 4 °C. Signals recorded 3 weeks post inoculation.

Features extracted from the sensor array responses to chemicals emitted by samples consisting of varying proportions of Fusarium-infected onion bulbs are shown in Figure 3.

Generally, the responses of the sensors increased with the higher ratio of the infected to non-infected onion bulbs. The highest signals were recorded for samples containing the highest number of infected bulbs stored at 25 °C, whereas samples with non-inoculated onion bulbs (0%), as well as samples stored at 4 °C, generated the lowest sensors’ signals.

**Figure 4:**
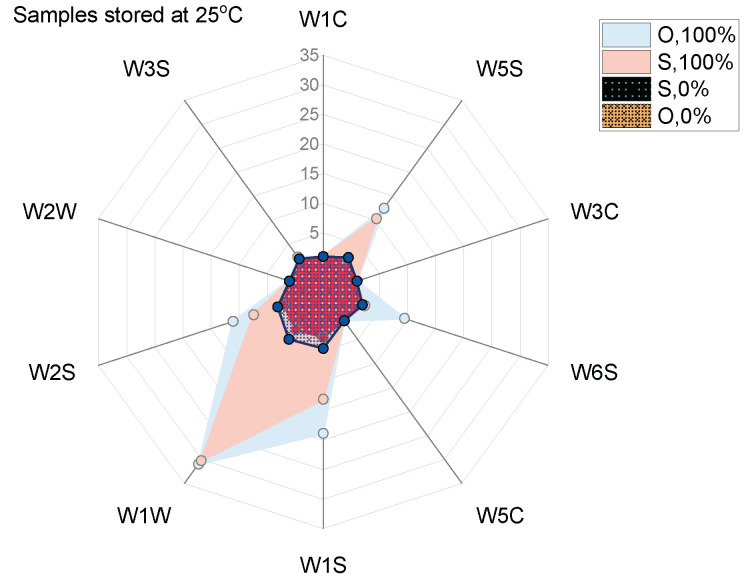
Polar plot of the volatile patterns emitted by non-infected onions and shallots (O, 0% and S, 0%, respectively) and diseased bulbs (O, 100%, and S, 100%) stored at 25 °C for 4 weeks post inoculation.

Sensor array response patterns to compounds released by healthy (0%) and infected (100%) onion and shallot bulbs are shown in Figure 4.

It was found that the patterns of sensors’ signals corresponding to samples containing only diseased onions and shallots were similar and significantly different from the patterns related to samples consisting of non-infected bulbs. All of the sensors’ signals were considerably lower for healthy bulbs’ samples in comparison to samples with diseased bulbs, where the signals were up to 30 G/G_0_.

**Figure 5:**
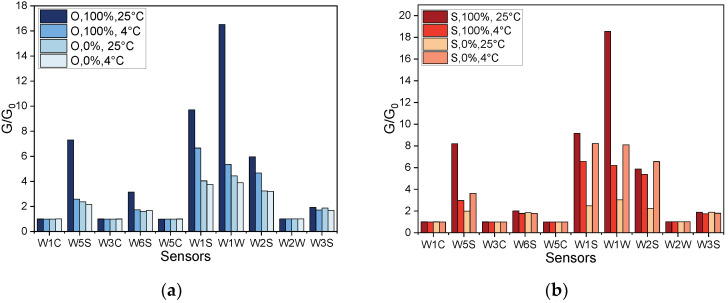
Sensors’ responses to samples consisting of healthy or Fusarium diseased bulbs stored at different temperatures for 2 weeks after inoculation. (**a**) Onion bulbs and (**b**) shallot bulbs.

The effect of the storage temperature on the emission of the volatiles was also evaluated based on sensors’ responses recorded 2 weeks post inoculation (Figure 5).

The sensor array’s responses were found to be significantly different depending on the analysed sample. The highest sensors’ signals were related to chemicals released by infected onion bulbs stored at 25 °C, while considerably lower responses were found from samples stored at 4 °C. Samples with non-infected onion bulbs regardless of temperature generated comparable sensors’ signals. On the other hand, sensors’ responses to chemicals emitted by samples containing non-infected and infected shallot bulbs stored at 4 °C were comparable. This suggests an absence or low degree of Fusarium infection in the case of shallots stored at 4 °C. Nevertheless, significant differences among sensors’ responses to volatile profiles of infected and non-infected shallot bulbs stored at 25 °C were observed.

### 3.3. PCA

**Figure 6:**
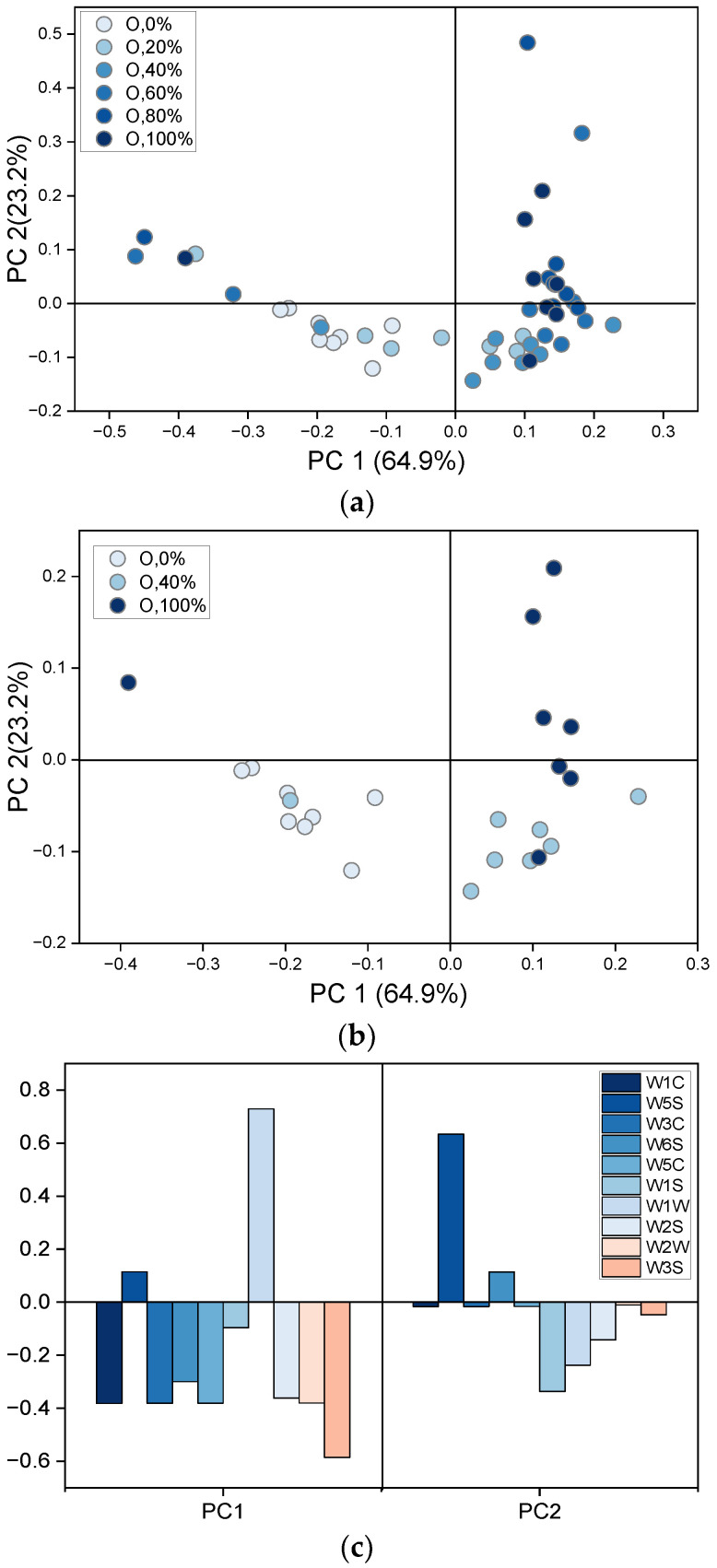
PCA score and loading plots illustrating volatile profiles’ patterns of samples consisting of infected and healthy onion bulbs at varying proportions. (**a**) Six groups of samples, (**b**) three groups of samples, and (**c**) loading plot.

Patterns of the sensors’ responses to compounds emitted by infected and non-infected shallot and onion bulbs were analysed using PCA. First, recorded signals from samples with varying proportions of infected and non-infected onion bulbs formed the datasets for PCA. The results of the analysis are presented in Figure 6.

The first two principal components explained 88% of the total variation. It can be seen that the position of the points on the graph is related to the type of sample. The score plot of the six groups of samples is shown in Figure 6a. The points corresponding to one week of bulbs’ storage at 25 °C are located close to each other and have the lowest PC1 value. Increasing storage time is reflected by points with increasing PC1 value. To clearly depict the trend of changes in odour profiles, three groups of samples were extracted from Figure 6a and are shown in Figure 6b.

Interestingly, the points corresponding to non-infected onion bulbs are located in one rectangle with PC1 < 0 and PC2 < 0. Differences in the position of the points representing healthy samples, over time, are the least, whereas they are the highest in the case of samples containing 100% infected bulbs. The calculated Euclidean distances between points representing the same type of sample over time provided a quantitative measure of these differences (data not shown). A correlation was found between the Euclidean distance between points corresponding to the same sample at the successive time points and the storage time. For most samples, the longer the storage time, the greater the distance between points on the PCA plot. The least difference was calculated for non-infected onion bulbs (0.16 in the PC1–PC2 space), while the highest was observed in the case of samples containing 60% and 80% of infected bulbs (0.68 and 0.66, respectively). Those results indicated that the volatile profiles of the samples with fewer infected bulbs changed significantly less than the volatile profiles of samples containing more infected bulbs over time. Therefore, the distance between samples could reflect the severity of the pathogenic fungi infection in the storage over time. Finally, the loading plot, presented in Figure 6c, revealed that all the sensors contributed significantly to the differences among samples. The highest contribution to PC1 showed sensor W1W, whereas sensor W5S contributed the most to PC2. Sensors W1C, W3C, and W5C demonstrated negligible contribution to PC2, but significantly high to PC1.

**Figure 7:**
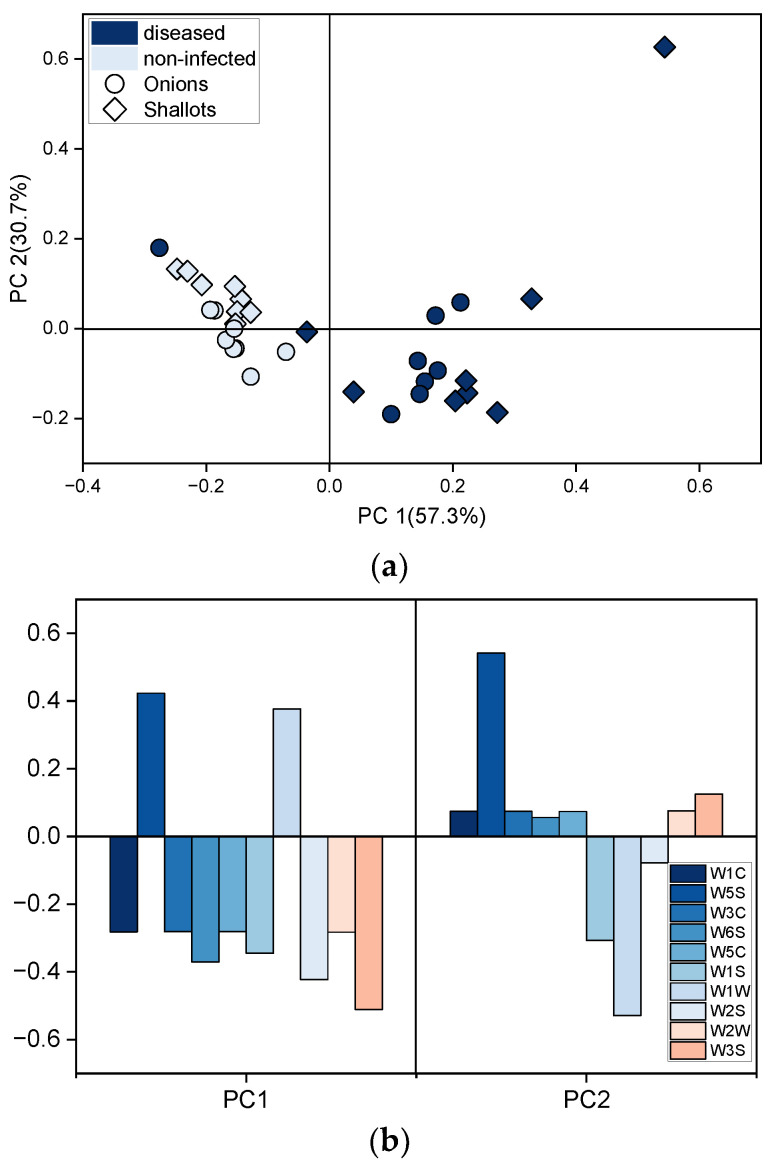
Volatile patterns of samples containing only non-infected (0%) or Fusarium infected (100%) onion or shallot bulbs stored at 25 °C. (**a**) Score plot based on sensor array responses recorded throughout experiment and (**b**) loading plot of the sensors.

Next, PCA was applied for visualizing the differences among samples containing healthy or 100% diseased onion or shallot bulbs stored at 25 °C. The PCA score plot based on the electronic nose’s responses is shown in Figure 7a.

The first two principal components captured 86% of the total variance of data. Moreover in this case, progress in the fungal infection can be associated with the points’ position on the plot. The points representing samples with non-infected bulbs formed one cluster characterized by the lowest PC1 values, while points related to infected samples significantly changed the position on the plot as storage time increased mainly along PC1. A clear distinction can be made among points corresponding to samples with infected and healthy onion and shallot bulbs in terms of PC1 values. However, points representing early fungal infection formed one cluster with the healthy ones. Nevertheless, based on the results it can be indicated that the electronic nose can be used to evaluate the progress of the fungal infection and to discriminate between healthy and diseased bulbs. Based on the sensors’ loading presented in Figure 7b, it can be seen that all of the sensors contributed to PC1, whereas three sensors showed high contribution to PC2, namely W5S, W1W, and W1S. Two of those sensors (W5S and W1W) demonstrated the highest input.

**Figure 8:**
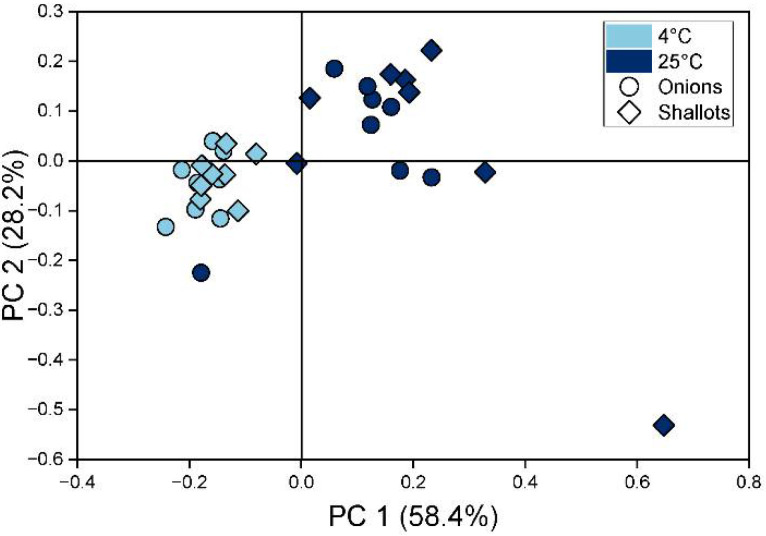
PCA score plot of infected onion and shallot bulbs stored at different temperatures.

The influence of the storage temperature on the volatile profiles was also evaluated using PCA. PCA was applied to data from sensors’ responses to infected onion and shallot bulbs stored at different temperatures (Figure 8).

The first two PCs explain 86% of the total variance in the dataset. Similar to Figure 7a, points corresponding to diseased onion and shallot bulbs stored at 4 °C form one cluster, while points representing fungal infection in bulbs kept at higher temperature form a second. With longer storage time, the points corresponding to samples stored at high temperature are characterised by higher values of PC1.

### 3.4. Classification of the Samples

The PCA results demonstrated that volatile patterns of the samples containing infected and non-infected onion and shallot bulbs at varying proportions significantly differ from each other as well as over time. Therefore, supervised chemometric methods were applied to:

1. Classify samples with onion bulbs in terms of the proportion of infected to healthy bulbs (3 classes), i.e., non-infected (0%), mildly diseased (20–40% infected bulbs), and severe diseased (60–100% infected bulbs);
2. Differentiate among samples containing non-infected (0%) and infected (100%) onion and shallot bulbs (two classes);

The pattern recognition methods, namely LDA, SVM, and k-NN, were employed to process datasets and their performance was compared based on the models’ accuracy, recall, and F1 score. To validate the classification ability of the models, a 5-fold cross-validation was used. Each of the algorithms was optimized through adjusting parameters including for LDA: number of feature and discriminant type; for SVM: kernel function, box constraints, kernel scale, and multiclass method; and for k-NN: number of neighbours, distance metric, and distance weight. Prior to the data processing, variables were auto scaled. The accuracy, recall, and F1 score of the developed models are shown in Table 2. The reported data is for the test set only.

All models succeeded in classification with most of the samples having accuracy between 66.7 and 96.9%, recall between 0.67 and 0.94, and F1 score between 0.74 and 0.97. LDA and optimized k-NN models showed the best performance with 89.6%, 0.9, and 0.92 accuracy, recall, and F1 score, respectively, in the case of differentiation among healthy, mildly, and severely diseased onion bulbs. K-NN optimization resulted in applying Chebyshev metric, 24 neighbours, and squared inverse distance weight. Significantly better classification abilities, with accuracy between 87.5 and 96.9%, showed models distinguishing between non-infected and 100% infected onion and shallots bulbs. SVM methods based on the third-degree polynomial kernel correctly classified samples with 96.9% accuracy, 0.94 recall, and 0.97 F1 score.

## 4. Discussion

This work explored the feasibility of using an electronic nose for detection and monitoring of basal rot disease development in onion and shallot bulbs artificially infected with *F. oxysporum* f. sp. *cepae*. The preliminary results indicated that the disease can be detected in both onion and shallot bulbs by the PEN 3 electronic nose. The sensor array responses to odours collected from samples with varying proportions of infected bulbs were found to be highly related to the type of sample. Higher signals were generated for samples consisting of a higher proportion of infected bulbs. These findings are in good accordance with the work of Wang et al. [7] where a relationship was observed between the total VOC emission rate of *F. oxysporum* f. sp. *cepae* -inoculated onions and the amount of fungal DNA contained in the bulb determined by PCR. In this study, five gas sensors including W5S, W6S, W1S, W1W, and W2S demonstrated the highest responses to all diseased samples as well as the highest signals’ variation. These sensors have been reported to be sensitive to nitrogen oxides, hydrogen, methane, sulphur organic compounds, alcohols, aromatic compounds, and a broad range of compounds (Table 1) [42,46]. Several publications have discussed volatile profiles of onions infected with *F. oxysporum* f. sp. *cepae* and identified characteristic VOCs related to basal rot disease using GC-MS. However, electronic noses, such as the one used in this study, are also capable of detecting small inorganic compounds, while GC-MS analysis is used for detection of a wider range of volatile compounds, especially with high molar mass. Therefore, these methods might be recognized as complementary approaches. In the study conducted by Wang et al. [7], 43 VOCs were detected in the volatile profiles of infected onion bulbs, among which 16 compounds were sulphur-organic compounds including 1-propanethiol, methyl propyl sulfide, dimethyl disulfide, styrene, and methyl propyl disulfide. Among them, 1-Propanethiol, methyl propyl sulfide, and styrene were suggested as the biomarker candidates with the highest association with fusarium basal rot disease due to a close correlation (r = 0.82–0.89) with the extent of infection evaluated for 7 weeks. In the previous work [59], several alcohols and esters were outlined as potential indicators of the disease. Vikram et al. [60] in their research also identified cyclopentane and high amounts of styrene as being associated with *Fusarium* infection. These findings are in line with the results of the electronic nose responses to chemicals emitted by *Fusarium*-infected bulbs presented here.

The differences in sensors’ responses to chemicals released by healthy and diseased onion samples suggest the feasibility of discrimination using EN technology. Differentiation between onions and shallots might be challenging due to similar patterns of sensors signals. Sensors generally described as selective to alcohols, sulphur-organic compounds, methane, and a broad range of organic substances demonstrated significant changes in responses to different health status samples. The sensor selective to sulphur-organic substances such as sulphides contributed to the differences in these patterns to the greatest degree. Furthermore, the lowest responses, comparable for all samples showed sensors W1C, W3C, W2W, and W3S, which are sensitive mostly to aromatic compounds, alkenes, and ammonia. Therefore, the volatile profiles of the studied samples included mostly Sulphur-based compounds, alcohols, and a broad range of organic compounds, although not aromatic ones. The results presented in Figure 3 might indicate that responses of the five sensors provide a sufficient amount of information on samples for their differentiation. On the other hand, the loading plots in Figure 6c and Figure 7b show that all sensors significantly contribute to PCA results, and reduction of the sensor array would not improve discrimination among samples. Sensors that have a low contribution to PC1 contributed highly to PC2 (or reversely) that capture 23–30% of the variance of the dataset, Figure 6c and Figure 7b, respectively.

The results presented here have also demonstrated that storage temperature significantly affects volatile profiles emitted by onion bulbs, with lower storage temperature inhibiting the progression of *Fusarium* infection and enabling the maintenance of healthy bulbs for a longer time. Furthermore, the results have also revealed that similar volatile profiles are released by onions and shallots with the same health status. This might indicate that several volatile compounds are specific to *Fusarium* infection and are emitted regardless of the host or that as onions and shallots are closely related, it may be the nature of the tissue structure. However, it was found that most of the sensors’ responses were slightly lower in the case of shallots.

Classification models based on LDA, SVM, and k-NN algorithms successfully differentiated among various rates of infected bulbs in the samples. The most straightforward methods, k-NN and LDA, showed the highest classification capabilities among three classes of samples. This might be related to the fact that k-NN algorithm is based on distance metric, which was similarly found on the PCA plots, and LDA algorithm is much similar to PCA. LDA applied to five sensors’ responses resulted in lower accuracy, recall, and F1 score, indicating that all sensors contributed to differences between samples.

Identification of healthy bulbs was successfully performed with all methods, with accuracy above 87.5% and just slight differences among accuracies of the models were observed. Nevertheless, SVM methods was found to demonstrate the best classification ability with accuracy 96.9% and 0.97 F1 score.

The results showed that sensor array responses to volatile profiles of the onion and shallot samples are a rich source of information which enables the identification of *Fusarium*-diseased samples as well as the evaluation of the proportion of diseased and healthy bulbs in a sample. Those tasks are of paramount importance for detection of basal rot in stores and a gas sensor array combined with pattern recognition methods can therefore be a useful tool for detecting and monitoring disease spread and progression. A limitation of this study is that it was undertaken in a laboratory environment with artificially infected bulbs. The next phase of our work will be to use an electronic nose in a store environment to see if the technology will work in a real-life scenario.

## 5. Conclusions

In this work, the PEN 3 electronic nose system was used for detection of *Fusarium oxysporum* f. sp. *cepae* in onion and shallot bulbs, monitoring the progression of basal rot development, and for discrimination between the varying proportions of diseased bulbs. To our best knowledge, this is the first report of a successful application of an electronic nose to detect a fungal infection in post-harvest onion and shallot bulbs. Sensor array responses to chemicals emitted by infected and non-infected bulbs combined with PCA and pattern recognition methods provided clear discrimination between healthy and diseased material as well as differentiation between samples with varying proportions of infected bulbs. All sensors had a significant contribution to the differentiation between samples. However, sensors sensitive to sulphur-organic compounds, nitrogen oxides, and a broad range of compounds contributed the highest degree to detection. The results of our preliminary studies proved the feasibility of the electronic nose to detect and monitor fungal infection and to recognize disease rate in bulb samples. Further studies are still needed with special attention paid on the early detection of disease. Electronic nose technology is constantly expanding, offering robust sensors with improved pattern recognition methods, and therefore it has great potential as a portable device for rapid, non-destructive monitoring of post-harvest crops’ health status.

## Figures and Tables

**Table 1:** General description of the PEN 3 electronic nose system’s sensors.

No	Producent’s Designation	General Description
1	W1C	aromatic compounds
2	W5S	broad range of compounds, nitrogen oxides
3	W3C	ammonia, aromatic compounds
4	W6S	Hydrogen
5	W5C	alkenes, aromatic compounds
6	W1S	methane, broad range of compounds
7	W1W	sulphur-organic compounds
8	W2S	alcohols, broad range of organic compounds
9	W2W	aromatic compounds, sulphur organic compounds
10	W3S	alkenes, aliphatic organic compounds

**Table 2:** Various supervised methods’ accuracy, recall, and F1 score applied to classify samples to three groups, namely non-infected, mild-infected, and severe diseased onion bulbs, and recognize non-infected and diseased onion and shallot bulbs.

	3 Class Classification			2 Class Classification		
	Accuracy	Recall	F1 Score	Accuracy	Recall	F1 Score
LDA	89.60%	0.90	0.92	93.80%	0.88	0.93
LDA 5 sensors	66.70%	0.67	0.74	90.60%	0.81	0.90
SVM linear	79.20%	0.79	0.84	87.50%	0.75	0.86
SVM optimized	87.50%	0.83	0.87	96.90%	0.94	0.97
k-NN optimized	89.60%	0.90	0.92	93.80%	0.88	0.93

Note: models with optimized parameters.

## Data Availability

Not applicable.

## Supplementary Materials

The following supporting information can be downloaded at: https://www.mdpi.com/article/10.3390/s22145453/s1, Figure S1: Visual symptoms of the fungal infection over time; Figure S2: Photos of the halved onion and shallot bulb stored for 31 days and 63 days post inoculation at 25 °C and 4 °C respectively; Figure S3: Sensors’ responses to chemicals collected from non-infected onion bulbs stored at 25 °C for 1.5 weeks post inoculation.
