# Supplementary material for: Preliminary Studies on Detection of Fusarium Basal Rot Infection in Onions and Shallots Using Electronic Nose
Source: Sensors (Basel). 2022 Jul 21;22(14):5453. doi: 10.3390/s22145453 (PMC9315870; doi:10.3390/s22145453)
Supplement: Supplementary file 1 [file sensors-22-05453-s001.zip › sensors-1799350-supplementary.pdf]

Supplementary material

Preliminary Studies on Detection of Fusarium Basal Rot Infection in Onions and Shallots Using Electronic Nose

Malgorzata Labanska [1,*], Sarah van Amsterdam [2], Sascha Jenkins [2], John P. Clarkson [2] and James A. Covington [3,*]

[1] The Plant Breeding and Acclimatization Institute-National Research Institute, Radzikow, 05-870 Blonie, Poland

[2] Warwick Crop Centre, School of Life Sciences, University of Warwick, Wellesbourne CV35 9EF, UK; sarah.van-amsterdam@warwick.ac.uk (S.v.A.); sascha.jenkins.1@warwick.ac.uk (S.J.); john.clarkson@warwick.ac.uk (J.P.C.)

[3] School of Engineering, University of Warwick, Coventry CV4 7AL, UK

* Correspondence: mwesoly@ch.pw.edu.pl or m.labanska@ihar.edu.pl (M.L.); j.a.covington@warwick.ac.uk (J.A.C.)

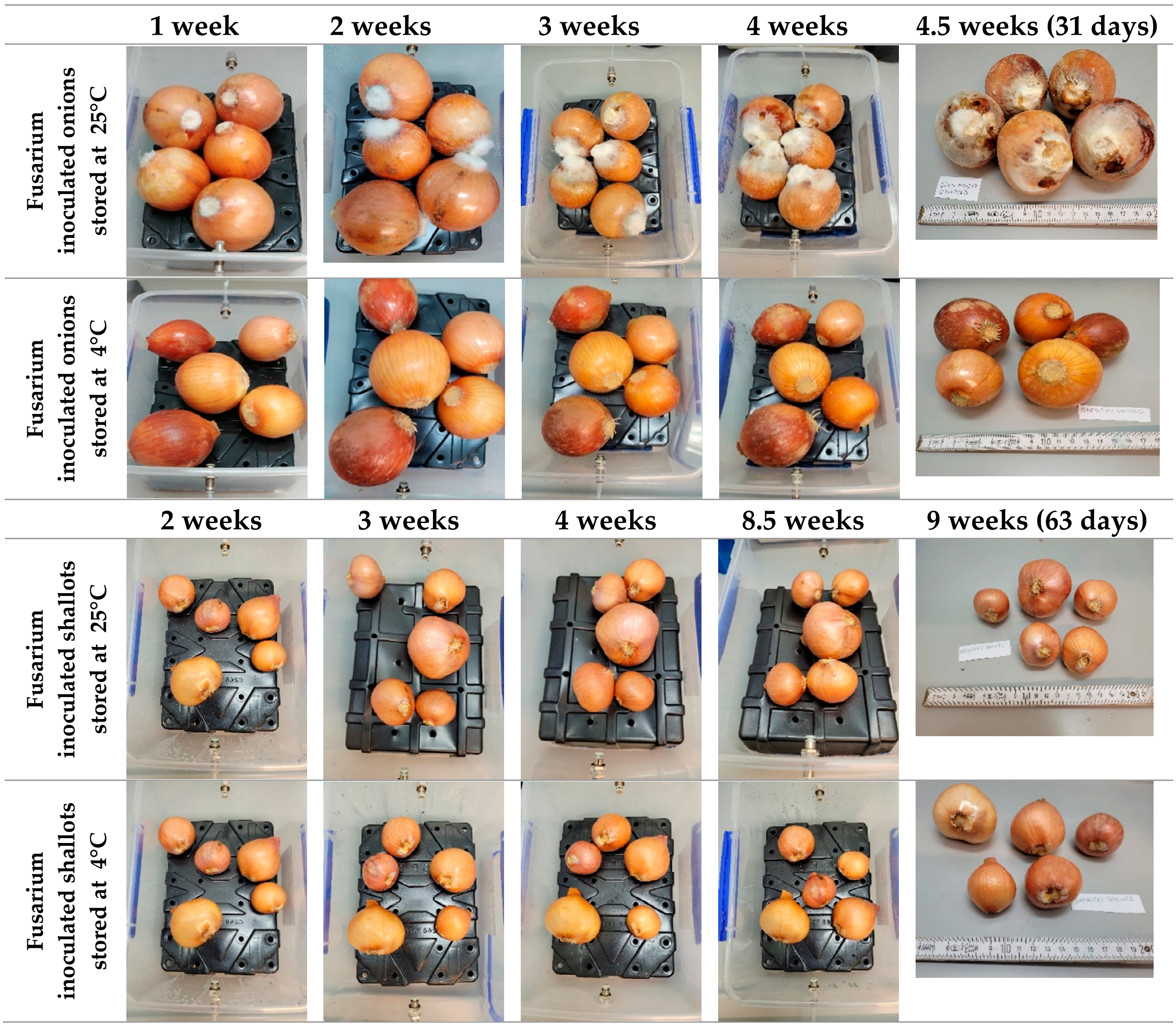

Figure S1. Visual symptoms of Fusarium basal rot development infection over time at 25°C and 4°C.

	25 °C	4 °C
**Onions inoculated with Fusarium**	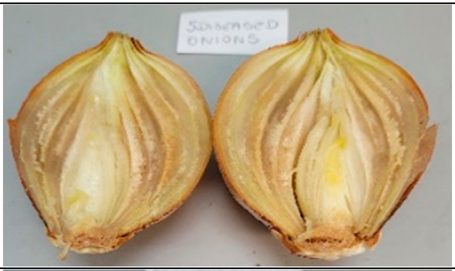	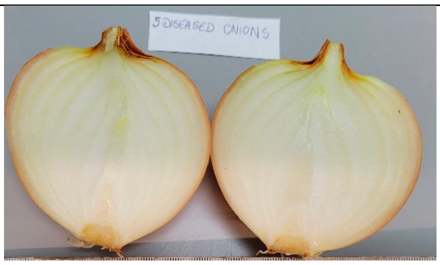
**Control onions (not inoculated)**	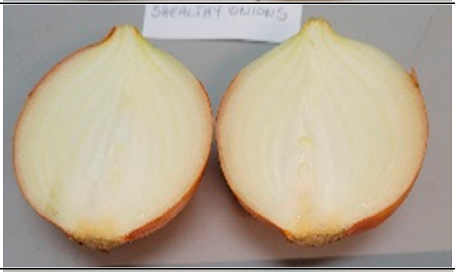	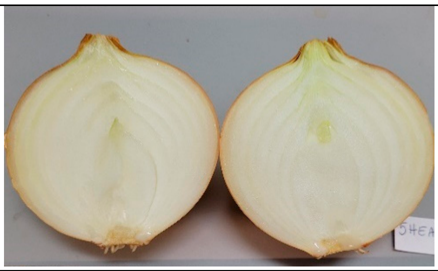
**Shallots inoculated with Fusarium**	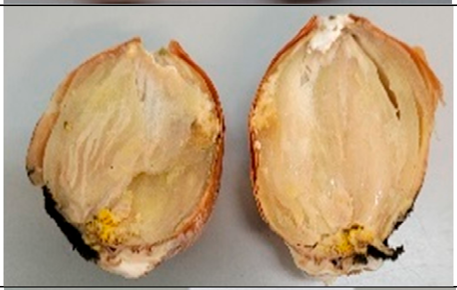	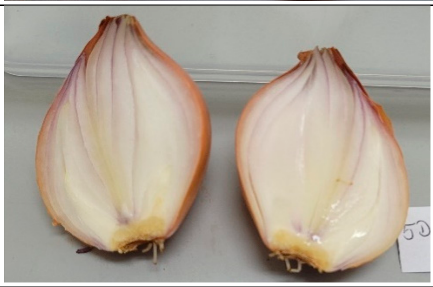
**Control shallots (not inoculated)**	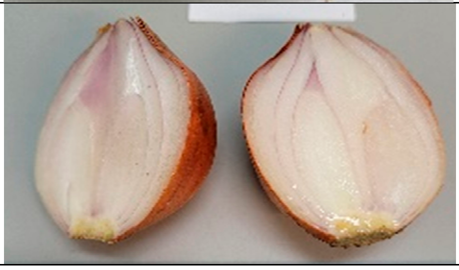	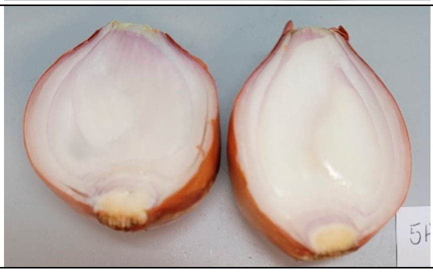

Figure S2. Exemplar onion and shallot bulbs for different treatments following dissection after 31 days and 63 days storage at 25 °C and 4 °C respectively. Severe internal basal rot symptoms observed for bulbs stored at 25 °C compared to an absence of any disease for bulbs stored at 4 °C and non-inoculated control bulbs.

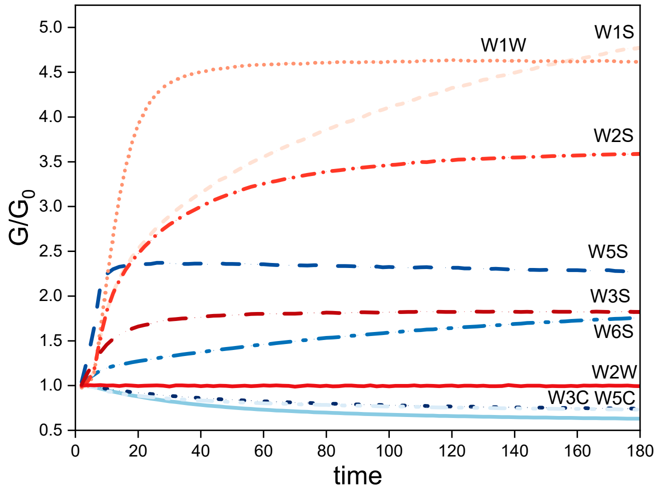

Figure S3. Sensors' responses to chemicals collected from non-infected onion bulbs stored at 25 °C for 1.5 weeks post inoculation.
